# Antiviral Treatment among Pregnant Women with Chronic Hepatitis B

**DOI:** 10.1155/2014/546165

**Published:** 2014-12-07

**Authors:** Lin Fan, Kwame Owusu-Edusei, Sarah F. Schillie, Trudy V. Murphy

**Affiliations:** National Center for HIV/AIDS, Viral Hepatitis, STD, and TB Prevention, Centers for Disease Control and Prevention, 1600 Clifton Road, MS G-37, Atlanta, GA 30333, USA

## Abstract

*Objective*. To describe the antiviral treatment patterns for chronic hepatitis B (CHB) among pregnant and nonpregnant women. *Methods*. Using 2011 MarketScan claims, we calculated the rates of antiviral treatment among women (aged 10–50 years) with CHB. We described the pattern of antiviral treatment during pregnancy and ≥1 month after delivery. *Results*. We identified 6274 women with CHB during 2011. Among these, 64 of 507 (12.6%) pregnant women and 1151 of 5767 (20.0%) nonpregnant women received antiviral treatment (*P* < 0.01). Pregnant women were most commonly prescribed tenofovir (73.4%) and lamivudine (21.9%); nonpregnant women were most commonly prescribed tenofovir (50.2%) and entecavir (41.3%) (*P* < 0.01). Among 48 treated pregnant women with an identifiable delivery date, 16 (33.3%) were prescribed an antiviral before pregnancy and continued treatment for at least one month after delivery; 14 (29.2%) started treatment during the third trimester and continued at least one month after delivery. *Conclusion*. Among this insured population, pregnant women with CHB received an antiviral significantly less often than nonpregnant women. The most common antiviral prescribed for pregnant women was tenofovir. These data provide a baseline for assessing changes in treatment patterns with anticipated increased use of antivirals to prevent breakthrough perinatal hepatitis B virus infection.

## 1. Introduction

An estimated 350 million individuals worldwide are chronically infected with hepatitis B virus (HBV) [1]. Perinatal HBV infection leads to chronic hepatitis B (CHB) in up to 90% of infants [2, 3]. About 25% of infants who develop CHB will die prematurely from cirrhosis, liver failure, and/or liver cancer [2–5]. Postexposure immunoprophylaxis consisting of hepatitis B vaccination and hepatitis B immune globulin (HBIG) can prevent up to 95% of perinatal HBV transmissions [2, 6]. Breakthrough perinatal HBV transmission occurs in 10–15% of infants born to pregnant women with high viral load even with postexposure immunoprophylaxis [3, 7].

The indications for antiviral treatment in CHB are based on the maternal serum viral load, liver enzyme (e.g., alanine aminotransferase) levels, hepatitis B e-antigen (HBeAg) status, liver histology, and HIV coinfection status [8]. The use of antivirals during pregnancy solely for prophylaxis of perinatal HBV transmission requires careful evaluation of potential risks and benefits among infants and pregnant women. Animal studies showed severe growth restriction and reduced bone mineral density among fetuses [9]. The development of drug resistance (particularly with lamivudine) and postpartum flare have been a concern for pregnant women receiving antiviral treatment during pregnancy. The major potential benefit of antiviral prophylaxis during pregnancy is to reduce viremia and decrease breakthrough perinatal HBV infections [10–13]. Antiviral treatment might also help manage maternal liver disease. With increasing data showing efficacy of antiviral prophylaxis to prevent perinatal HBV transmission, and an acceptable safety profile for pregnant women and infants, more experts and organizations are recommending consideration of prophylactic antiviral treatment starting in the third trimester for pregnant women with high viral load (e.g., ≥10^6^ IU/mL) to prevent breakthrough perinatal HBV transmission [1, 8, 11–19].

None of the Food and Drug Administration (FDA)-approved antiviral agents for CHB is classified as pregnancy category A [14, 16, 17]. However, experience with some agents (e.g., lamivudine and tenofovir disoproxil fumarate [tenofovir]) has been extensive among HIV-infected pregnant women [20]. Of five oral antiviral agents available for treatment of CHB, telbivudine and tenofovir are classified as FDA Pregnancy Category B (animal studies indicate no fetal risk, but no humans studies exist, or adverse effects in animals but not in humans), and lamivudine, adefovir dipivoxil (adefovir), and entecavir are classified as Pregnancy Category C (no adequate human or animal studies; benefit may outweigh risk) [14, 17]. Information on the use of antivirals for CHB during pregnancy in the United States of America (USA) is scarce. The objective of this study was to describe antiviral treatment among pregnant and nonpregnant women with CHB in 2011 using claims data from MarketScan, with a focus on treatment patterns among the pregnant women.

## 2. Methods

### 2.1. Data Source

We examined inpatient, outpatient, and drug claims data from the 2011 Truven Health MarketScan Commercial Claims and Encounters Databases (Truven Health MarketScan Databases, Truven Health Analytics, Ann Arbor, MI), which collects data from nationwide employer-sponsored health insurance plans on more than 50 million covered USA individuals [21]. The MarketScan claims databases capture the full continuum of care in all settings including outpatient and inpatient visits. MarketScan drug claims contain detailed information on outpatient prescriptions [21]. The study did not require human subjects review because the analysis employed secondary data without personal identifiers (Federal Regulations at 45 CFR part 46).

### 2.2. Study Population

We restricted the study sample to women aged 10–50 years to capture a broad range of ages when women might have been pregnant in the USA. We classified women as having CHB when the ICD-9 code was 07022, 07023, 07032, or 07033 (Table 1). Because the antiviral drugs for human immunodeficiency virus (HIV) can overlap with antivirals used to treat HBV, we also identified women who might be coinfected with HIV. We classified women as coinfected with HIV when the ICD-9 code was 042, V08, 79571, or 07953 (Table 1). For women with CHB, we extracted data on age in 2011, on residence by metropolitan or nonmetropolitan area, and by region of the USA (Northeast, North Central, South, and West). We identified pregnant women among women with CHB if they had a delivery code in 2011 (Table 1). We defined antiviral treatment for CHB by having national drug codes for one or more of the 5 oral antiviral drugs for CHB. We examined antiviral treatment in 2010, in addition to 2011, to capture antiviral treatment before and throughout pregnancy.

**Figure 1:**
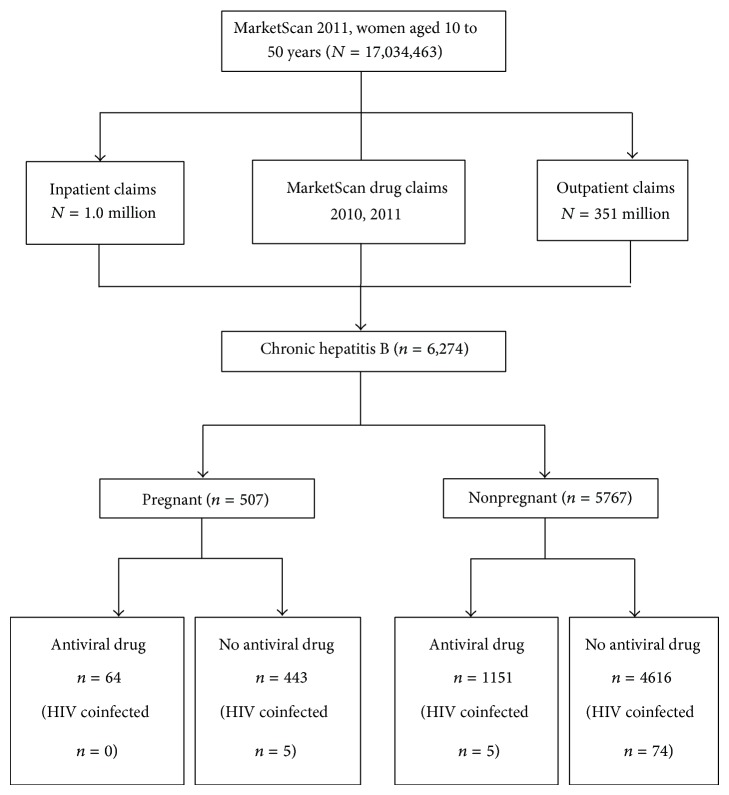
Selection of subjects.

For pregnant women, we identified the delivery dates and then defined a pregnancy period as 3 trimesters with 13 weeks (91 days) in each trimester (Figure 1). Pregnant women without a delivery date were excluded from evaluation of treatment patterns. We looked for prescriptions of antiviral drugs for up to 300 days before the delivery date and extracted the antiviral drug prescription dates and supply. We assumed if a prescription for an antiviral was filled, the woman was receiving antiviral treatment. We then examined treatment patterns, describing the time the antiviral prescription was filled relative to the delivery date, the duration of treatment, and the daily cost of treatment for each antiviral drug.

### 2.3. Analyses

We calculated the frequencies of pregnant women with CHB who were prescribed antiviral treatment and the frequencies with which various antiviral drugs were prescribed. We calculated the mean cost per day for each antiviral drug. Using Pearson chi-square test and logistic regression, we assessed the association between antiviral treatment and age and between antiviral treatment and pregnancy status. We further determined whether the choice of antiviral differed among pregnant and nonpregnant women with CHB, and if there was regional variation (by metropolitan status and by the USA census region) in antiviral treatment. The data were analyzed using SAS (version 9.3; SAS Institute, Cary, NC).

## 3. Results

The 2011 database contained records of more than 17 million women aged 10–50 years; among these, 6,274 women were classified as having CHB. Among women with CHB, 507 (8.1%) were identified as pregnant (Figure 1). Compared to nonpregnant women, pregnant women were younger (mean 33.0 years versus 37.5 years, resp., *P* < 0.01) and less likely to receive antiviral treatment (12.6% versus 20.0%, resp., *P* < 0.001). Among pregnant women, no significant difference was found between treated and untreated women by age, residence in a metropolitan area, or region of residence in the USA (Table 2). Among nonpregnant women, treated women were more likely than untreated women to be slightly older (mean 38.8 versus 37.2 years, *P* < 0.01) and to live in the South (31.5% versus 25.2%, *P* < 0.01) and less likely to live in the Northeast (19.5% versus 23.0%, *P* < 0.01) and to be coinfected with HIV (0.4% versus 1.6%, *P* < 0.01). Treatment status did not differ significantly by metropolitan residence area (Table 2). Results from a logistic regression analysis showed that older age (OR: 1.02, 95% confidence interval (95% CI): 1.01–1.03) and living in the South compared to in the Northeast (OR: 1.42, 95% CI: 1.19–1.70) were positively associated with antiviral treatment; being pregnant (OR: 0.66, 95%: 0.51–0.87) or coinfected with HIV (OR: 0.28, 95% CI: 0.11–0.70) was negatively associated with antiviral treatment.

For 1215 women receiving treatment, the most frequently prescribed antivirals were tenofovir (51.4%) and entecavir (39.8%); 88 (7.2%) women received more than one antiviral during the study period. Receipt of two or more antivirals was more common among pregnant than nonpregnant women (15.6% versus 6.8% resp., *P* < 0.01) (Table 3). The proportion of women with CHB who received lamivudine and tenofovir was higher for pregnant than nonpregnant women, and the proportion who received entecavir was lower for pregnant than nonpregnant women (*P* < 0.01). The mean cost of antiviral treatment per day was the highest for adefovir ($32) and the lowest for lamivudine ($10) (Table 3).

We identified delivery dates in 2011 for 48 of 64 pregnant women receiving antiviral treatment. Treatment started before pregnancy and continued for at least one month after delivery in 16 (33.3%) women. Treatment began during the third trimester for 14 (29.2%) pregnant women and was continued more than one month after delivery for 11 (78.6%) women. Antiviral treatment prior to pregnancy was terminated for 8 women who became pregnant (Table 4). The most commonly prescribed antiviral drugs among pregnant women were tenofovir (66.7%, 32 of 48 pregnant women) and lamivudine (27.1%, 13 of 48 pregnant women).

We identified 84 (1.3%) women with CHB coinfected with HIV; 5 were pregnant. We identified 5 women with HIV coinfection prescribed antiviral drugs; none were pregnant. Three of the women were prescribed tenofovir and two women were prescribed adefovir dipivoxil.

## 4. Discussion

An increasing number of reports provide guidance on treating pregnant women with CHB [16, 18], and many cite the accumulating evidence for the efficacy of antivirals to enhance prevention of perinatal HBV transmission among pregnant women with high viral load [11–13, 16, 18]. Few studies have assessed the use of antivirals for pregnant women with CHB [22]. Our analysis of MarketScan data showed that 12.6% of pregnant women with CHB who delivered in 2011 received antiviral treatment during 2010 and 2011. In contrast, 20.0% of nonpregnant women received antiviral treatment during 2010 and 2011. The significantly lower use of antiviral treatment among pregnant compared to nonpregnant women was consistent with safety concerns for use of antiviral drugs during pregnancy [1, 8, 14, 16]. About one-third of the pregnant women receiving antiviral treatment for CHB had been receiving treatment before pregnancy. Pregnant women with significant liver disease and viremia are recommended to receive close monitoring for postpartum hepatic flare, which is most often manifested by an elevation of serum alanine aminotransferase [1, 16]. Of the 29% of pregnant women who started treatment during the third trimester, most continued receiving an antiviral for more than one month after delivery, suggesting that an antiviral was prescribed for ongoing severe liver disease rather than for prophylaxis to prevent perinatal HBV transmission [8].

The most commonly used antivirals prescribed for pregnant women were tenofovir and lamivudine. Tenofovir was FDA-approved for treatment of CHB in 2008 and has shown very low or no resistance by hepatitis B virus after prolonged use [11, 23]. Lamivudine was FDA-approved for treatment of CHB in 1998 and is the most studied antiviral agent in pregnancy [1]. We found a higher proportion of pregnant women were prescribed lamivudine compared to nonpregnant women. Short-term use of lamivudine in the last trimester is reported to be safe and is accompanied by a low risk of developing drug resistance [5, 13]. Long-term use of lamivudine is associated with development of HBV resistance [3, 11, 14, 23].

Some experts recommend deferring antiviral treatment for women who are planning a pregnancy unless they have active or advanced liver disease [8, 16]. If a patient with CHB becomes pregnant during treatment, the treatment indications can be reevaluated and the choice of antiviral agent reconsidered. If an antiviral agent is continued during pregnancy, tenofovir generally has been the agent of choice because of its effectiveness, low resistance rate, and safety profile during pregnancy. We found that treatment was terminated at some point before delivery for about one-fifth of the women who became pregnant during antiviral treatment; the rates of change to a different antiviral also were higher among pregnant than nonpregnant women [16].

Women with positive e antigen or high viral load have greater risk for transmitting HBV to their infant, even when the infant receives postexposure immunoprophylaxis [1, 14, 15, 17]. An increasing number of studies have shown that maternal antiviral prophylaxis during pregnancy can suppress HBV replication and might reduce up to 70% of perinatal HBV transmissions compared to postexposure immunoprophylaxis alone (HBIG and hepatitis B vaccination) [11–13]. Reports include use of lamivudine, telbivudine, and tenofovir for maternal prophylaxis [11–13]. Although concerns have been raised about the safety of antiviral treatment or prophylaxis during pregnancy, increasing evidence suggests that the incidences of adverse events among pregnant women and infants receiving antiviral treatment for CHB initiated during the last trimester are comparable to those of women and infants without antiviral treatment [9, 11–14, 16, 24]. In practice, when prevention of perinatal transmission of HBV is the purpose of prescribing antivirals, prophylaxis is initiated during the last trimester and stopped soon after delivery [1, 14, 25, 26].

Previous studies have shown the economic value of antiviral treatment for CHB during pregnancy, if used selectively to reduce the risk of perinatal HBV [3, 5, 27]. We compared the cost-effectiveness of five strategies and showed that antiviral treatment during the last trimester for women with high viral load, either after positive HBeAg screening or viral load testing, was cost-effective compared to the current active-passive immunoprophylaxis strategy alone [27]. Given the increasing evidence for the efficacy, safety, and economic value of antiviral prophylaxis during pregnancy, in 2012 the European Association for the Study of the Liver (EASL) and the Asian Pacific Association for the Study of the Liver (APASL) recommended considering a prophylactic antiviral in the third trimester for pregnant women with high viral load. Notably, this strategy is not a replacement for active-passive immunoprophylaxis for infants known to be at risk for perinatal HBV transmission, and universal infant hepatitis B vaccination, which should be continued. The results from our study indicate that antiviral prophylaxis during pregnancy to prevent perinatal transmission was uncommon in 2011 among women with employer-sponsored health insurance.

Although clinical and laboratory data are important to define disease severity and the need for treatment, our MarketScan database lacked this information [8, 15, 17, 20]. Without clinical and laboratory data, we were unable to examine for differences in the indications for antiviral treatment among pregnant and nonpregnant women. A limitation of using MarketScan medical claims data for insurance reimbursement is that these data are not representative of the USA population. MarketScan consists of a convenience sample of individuals with private employer insurance. For example, the prevalence of CHB among pregnant women in our study was 0.05%, considerably lower than the estimated 0.3–0.8% prevalence overall in the USA population [4, 28]. Patients with CHB who did not receive private employer insurance and patients with CHB who did not have a billable medical intervention would not have been identified in the analysis. Patients with insurance who purchased antiviral drugs would have drug claims data indicating CHB and, therefore, the treatment patterns likely were representative of the population that was covered by private insurance. Pregnant women with other forms of medical coverage (e.g., military) or with no insurance might vary in antiviral treatment patterns for CHB because of different capacities to access health care and antiviral drugs.

This study investigated the use of antiviral drugs among pregnant women with CHB infection who have private insurance. We found that a lower proportion of pregnant than nonpregnant women received antiviral treatment in 2011, and differences existed in the type of prescribed antivirals between pregnant and nonpregnant women. The most commonly prescribed antiviral for both pregnant and nonpregnant women was tenofovir in this population of employer sponsored insurance participants. These data provide a baseline evaluation of the use of antivirals in pregnant women with severe CHB disease. This information might be of particular interest if additional evidence of the safety and efficacy of HBV antiviral prophylaxis during pregnancy support its use as an adjunct to postexposure prophylaxis for the prevention of perinatal HBV transmission among the subsets of pregnant women with high viral load [1, 11–18].

## Figures and Tables

**Table 1:** List of international classification of diseases, 9th revision, clinical modification (ICD-9-CM), and current procedural terminology (CPT) codes for chronic hepatitis B, pregnancy, delivery, and HIV.

ICD-9-CM/CPT codes	Code description
Hepatitis B	
ICD-9-CM 070.22	Chronic hepatitis B with hepatic coma without hepatitis delta
ICD-9-CM 070.23	Chronic hepatitis B with hepatic coma with hepatitis delta
ICD-9-CM 070.32	Chronic hepatitis B without mention of hepatic coma without mention of hepatitis delta
ICD-9-CM 070.33	Chronic hepatitis B without mention of hepatic coma with hepatitis delta

HIV	
ICD-9-CM 042	Human immunodeficiency virus
ICD-9-CM V08	Asymptomatic HIV infection status
ICD-9-CM 795.71	Nonspecific serologic evidence of HIV
ICD-9-CM 079.53	HIV, type 2

Pregnancy and delivery	
ICD-9-CM 650	Normal delivery
ICD-9-CM 658	Other problems associated with amniotic cavity and membranes
ICD-9-CM 659	Other indications for care or intervention related to labor and delivery not elsewhere classified
ICD-9-CM 660–669	Complications occurring mainly in the course of labor and delivery
ICD-9-CM 670–677	Complications of the puerperium
ICD-9-CM V24.0	Postpartum care and examination immediately after delivery
ICD-9-CM V27	Outcome of delivery
ICD-9-CM procedure 69.02	Dilation and curettage following delivery or abortion
ICD-9-CM procedure 69.52	Aspiration curettage following delivery or abortion
ICD-9-CM procedure 72	Forceps, vacuum, and breech delivery
ICD-9-CM procedure 73	Other procedures inducing or assisting delivery
ICD-9-CM procedure 74	Cesarean section and removal of fetus
ICD-9-CM procedure 75.5	Repair of current obstetric laceration of uterus
ICD-9-CM procedure 75.6	Repair of other current obstetric lacerations
ICD-9-CM procedure 75.7	Manual exploration of uterine cavity, postpartum
ICD-9-CM procedure 75.8	Obstetric tamponade of uterus or vagina
CPT 01958, 01960-2, 01967-9	Anesthesia for delivery
CPT 59200	Insertion of cervical dilator
CPT 59300	Episiotomy or vaginal repair
CPT 59400–59414	Vaginal delivery
CPT 59510, 59514	Cesarean section delivery
CPT 59610, 59612, 59618, 59620	Delivery after previous cesarean delivery

**Table 2:** Characteristics of pregnant women with chronic hepatitis B virus infection in 2011 by prescription of antiviral treatment.

	Pregnant women (*N* = 507)				Nonpregnant women (*N* = 5767)^*^			
	Antiviral treatment		No antiviral treatment		Antiviral treatment		No antiviral treatment	
	(*n* = 64)		(*n* = 443)		(*n* = 1151)		(*n* = 4616)	
Mean age (range)	33.4 (22–46) years		32.9 (14–48) years		38.8 (10–50) years		37.2 (10–50) years	

	*N*	%	*N* ^†^	%	*N* ^†^	%	*N* ^†^	%

Metropolitan								
Yes	62	96.9	430	97.1	1115	96.9	4484	97.1
No	2	3.1	12	2.7	36	3.1	132	2.9
Region								
Northeast	15	23.4	101	22.8	225	19.5	1063	23.0
North Central	10	15.6	77	17.4	129	11.2	596	12.9
South	14	21.9	99	22.3	362	31.5	1163	25.2
West	25	39.1	156	35.2	426	37.0	1722	37.3
HIV coinfection								
Yes	0	0	5	1.1	5	0.4	74	1.6
No	64	100.0	438	98.9	1146	99.6	4542	98.4

^*^The differences between nonpregnant women with and without antiviral treatment were statistically significant for age, region, and HIV status at *P* < 0.01.

^†^Percentages were of total subjects: due to missing or invalid data, the sum of some subcategories and their associated percentages do not equal 100.

**Table 3:** Proportion and types of antiviral agents received by women age 10–50 years with chronic hepatitis B virus infection.

	Total^*^ (*N* = 6274)		Nonpregnant (*N* = 5767)		Pregnant (*N* = 507)		Pregnant versus nonpregnant	Cost ($/day)
	*N*	%^†^	*N*	%^†^	*N*	%^†^	*P* value	
Antiviral prescribed	1215	19.4	1151	20.0	64	12.6	<0.01	
Antiviral							<0.01	
Adefovir dipivoxil	102	8.4	99	8.6	3	4.7	0.357	32
Entecavir	483	39.8	475	41.3	8	12.5	<0.01	25
Lamivudine	73	6.0	59	5.1	14	21.9	<0.01	10
Telbivudine	20	1.6	18	1.6	2	3.1	0.284	22
Tenofovir disoproxil fumarate	625	51.4	578	50.2	47	73.4	<0.01	25
Two or more drugs	88	7.2	78	6.8	10	15.6	0.020	—

^*^Among 84 women coinfected with HIV and chronic hepatitis B virus infection, 5 (6.0%) were prescribed an antiviral, 2 (40%) adefovir dipivoxil, and 3 (60%) tenofovir disoproxil. None of the 5 coinfected women who received an antiviral was pregnant.

^†^Percentages total more than 100% because some women received more than one antiviral.

**Table 4:** Antiviral treatment patterns among 48 pregnant women with chronic hepatitis B virus infection with a delivery date in 2011.

	Before delivery	1st trimester	2nd trimester	3rd trimester	Post <1 m	Post >1 m
*N* = 2	✓					

*N* = 4	✓	✓				

*N* = 2	✓	✓	✓			

*N* = 3^*^	✓	✓	✓	✓		

*N* = 16	✓	✓	✓	✓	✓	✓

*N* = 1^*^		✓	✓	✓	✓	

*N* = 1		✓	✓	✓	✓	✓

*N* = 2			✓	✓	✓	

*N* = 1^†^			✓	✓	✓	✓

*N* = 1				✓		

*N* = 2				✓	✓	

*N* = 11^†^				✓	✓	✓

*N* = 2						✓

No HIV coinfected pregnant women received antiviral treatment.

^*^No data for 2012 were available to determine if the woman continued to receive antiviral treatment.

^†^No data in 2010 were available to determine if the woman received antiviral treatment starting at an earlier time.

## References

[1] Giles M. L., Visvanathan K., Lewin S. R., Sasadeusz J. (2012). Chronic hepatitis B infection and pregnancy. *Obstetrical and Gynecological Survey*.

[2] Margolis H. S., Coleman P. J., Brown R. E., Mast E. E., Sheingold S. H., Arevalo J. A. (1995). Prevention of hepatitis B virus transmission by immunization: an economic analysis of current recommendations. *The Journal of the American Medical Association*.

[3] Unal E. R., Lazenby G. B., Lintzenich A. E., Simpson K. N., Newman R., Goetzl L. (2011). Cost-effectiveness of maternal treatment to prevent perinatal hepatitis B virus transmission. *Obstetrics and Gynecology*.

[4] Din E. S., Wasley A., Jacques-Carroll L., Sirotkin B., Wang S. (2011). Estimating the number of births to hepatitis B virus-infected women in 22 states, 2006. *Pediatric Infectious Disease Journal*.

[5] Nayeri U. A., Werner E. F., Han C. S., Pettker C. M., Funai E. F., Thung S. F. (2012). Antenatal lamivudine to reduce perinatal hepatitis B transmission: a cost-effectiveness analysis. *The American Journal of Obstetrics and Gynecology*.

[6] Lee C., Gong Y., Brok J., Boxall E. H., Gluud C. (2006). Effect of hepatitis B immunisation in newborn infants of mothers positive for hepatitis B surface antigen: systematic review and meta-analysis. *British Medical Journal*.

[7] Zou H., Chen Y., Duan Z., Zhang H., Pan C. (2012). Virologic factors associated with failure to passive-active immunoprophylaxis in infants born to HBsAg-positive mothers. *Journal of Viral Hepatitis*.

[8] Yapali S., Talaat N., Lok A. S. (2014). Management of hepatitis B: our practice and how it relates to the guidelines. *Clinical Gastroenterology and Hepatology*.

[9] Wang L., Kourtis A. P., Ellington S., Legardy-Williams J., Bulterys M. (2013). Safety of tenofovir during pregnancy for the mother and fetus: a systematic review. *Clinical Infectious Diseases*.

[10] Dienstag J. L., Schiff E. R., Wright T. L., Perrillo R. P., Hann H.-W. L., Goodman Z., Crowther L., Condreay L. D., Woessner M., Rubin M., Brown N. A. (1999). Lamivudine as initial treatment for chronic hepatitis B in the United States. *The New England Journal of Medicine*.

[11] Pan C. Q., Mi L.-J., Bunchorntavakul C. (2012). Tenofovir disoproxil fumarate for prevention of vertical transmission of hepatitis b virus infection by highly viremic pregnant women: a case series. *Digestive Diseases and Sciences*.

[12] Pan C. Q., Han G.-R., Jiang H.-X., Zhao W., Cao M.-K., Wang C.-M., Yue X., Wang G.-J. (2012). Telbivudine prevents vertical transmission from HBeAg-Positive women with chronic hepatitis B. *Clinical Gastroenterology and Hepatology*.

[13] Shi Z., Yang Y., Ma L., Li X., Schreiber A. (2010). Lamivudine in late pregnancy to interrupt in utero transmission of hepatitis B virus: a systematic review and meta-analysis. *Obstetrics & Gynecology*.

[14] Buchanan C., Tran T. T. (2010). Management of chronic hepatitis B in pregnancy. *Clinics in Liver Disease*.

[15] Degli Esposti S., Shah D. (2011). Hepatitis B in pregnancy: challenges and treatment. *Gastroenterology Clinics of North America*.

[16] European Association for the Study of the Liver (2013). EASL clinical practice guidelines: management of chronic hepatitis B virus infection. *Journal of Hepatology*.

[17] Petersen J. (2011). HBV treatment and pregnancy. *Journal of Hepatology*.

[18] Liaw Y. F., Kao J. H., Piratvisuth T. (2012). Asian-Pacific consensus statement on the management of chronic hepatitis B: a 2012 update. *Hepatology International*.

[19] Tong M. J., Pan C. Q., Hann H.-W., Kowdley K. V., Han S.-H. B., Min A. D., Leduc T.-S. (2011). The management of chronic hepatitis B in Asian Americans. *Digestive Diseases and Sciences*.

[20] Doherty M., Ford N., Vitoria M., Weiler G., Hirnschall G. (2013). The 2013 WHO guidelines for antiretroviral therapy: evidence-based recommendations to face new epidemic realities. *Current Opinion in HIV and AIDS*.

[21] Thomson Reuters (2011). *MarketScan Databases User Guide and Database Dictionary*.

[22] Ahn J., Salem S. B., Cohen S. M. (2010). Evaluation and management of hepatitis B in pregnancy: a survey of current practices. *Gastroenterology and Hepatology*.

[23] Woo G., Tomlinson G., Nishikawa Y., Kowgier M., Sherman M., Wong D. K. H., Pham B., Ungar W. J., Einarson T. R., Heathcote E. J., Krahn M. (2010). Tenofovir and entecavir are the most effective antiviral agents for chronic hepatitis B: a systematic review and Bayesian meta-analyses. *Gastroenterology*.

[24] Xiao X. M., Li A. Z., Chen X., Zhu Y. K., Miao J. (2007). Prevention of vertical hepatitis B transmission by hepatitis B immunoglobulin in the third trimester of pregnancy. *International Journal of Gynecology and Obstetrics*.

[25] Honkoop P., De Man R. A., Niesters H. G. M., Zondervan P. E., Schalm S. W. (2000). Acute exacerbation of chronic hepatitis B virus infection after withdrawal of lamivudine therapy. *Hepatology*.

[26] ter Borg M. J., Leemans W. F., de Man R. A., Janssen H. L. A. (2008). Exacerbation of chronic hepatitis B infection after delivery. *Journal of Viral Hepatitis*.

[27] Fan L., Owusu-Edusei K., Schillie S. F., Murphy T. V. (2014). Cost-effectiveness of testing Hepatitis B-positive pregnant women for Hepatitis B e antigen or viral load. *Obstetrics and Gynecology*.

[28] Wasley A., Kruszon-Moran D., Kuhnert W., Simard E. P., Finelli L., McQuillan G., Bell B. (2010). The prevalence of hepatitis B virus infection in the United States in the era of vaccination. *Journal of Infectious Diseases*.

